# Elevated lactate dehydrogenase-to-albumin ratio: a novel and independent predictor of ventricular aneurysm in STEMI patients

**DOI:** 10.3389/fendo.2026.1775998

**Published:** 2026-05-13

**Authors:** Dong Hu, Ting Huang, Dongyang Wu, Qinshuo Zhao, Kai Zhang, Yuanlin Zou, Xin Guo, Yi Zhou

**Affiliations:** 1 Department of Cardiology, The Central Hospital of Wuhan, Tongji Medical College, Huazhong University of Science and Technology, Wuhan, China; 2 Key Laboratory for Molecular Diagnosis of Hubei Province, The Central Hospital of Wuhan, Tongji Medical College, Huazhong University of Science and Technology, Wuhan, China; 3 Hubei Provincial Engineering Research Center of Intestinal Microecological Diagnosis and Treatment Technology and Clinical Application, Wuhan, Hubei, China; 4 Department of Cardiology, Renmin Hospital of Wuhan University, Wuhan, China; 5 People’s Hospital of Zhengzhou University, Fuwai Central China Cardiovascular Hospital, Henan Provincial People’s Hospital, Zhengzhou, Henan, China

**Keywords:** acute ST-segment elevation myocardial infarction, lactate dehydrogenase to albumin ratio, left ventricular aneurysm, predictor, risk

## Abstract

**Background:**

Left ventricular aneurysm (LVA) is a major mechanical complication following acute ST-segment elevation myocardial infarction (STEMI). This study aimed to evaluate the predictive ability of the lactate dehydrogenase to albumin ratio (LAR) for the risk of LVA among STEMI patients.

**Methods:**

The study was conducted from July 2018 to 2025 across three medical centers in China. The study cohort comprised STEMI patients who underwent primary percutaneous coronary intervention across three medical centers. Multivariate logistic regression analysis and Restricted cubic spline plots were employed to assess the association between different LAR levels and LVA risk. Subgroup analyses were conducted to assess the consistency of the result.

**Results:**

The study included 551, 471, and 1384 eligible participants from the first, second, and third cohort, respectively. After adjusting for potential confounders, individuals in the highest quartile of LAR (Q4) demonstrated a significantly elevated risk of developing LVA compared to those in the lowest quartile (Q1) across all cohorts (First cohort: OR = 3.63, 95% CI = 1.65 - 7.96, P = 0.001; Second cohort: OR = 6.01, 95% CI = 2.22 - 16.26, P< 0.001; Third cohort: OR = 2.41, 95% CI = 1.47 - 3.96, P< 0.001). RCS analysis revealed a positive linear association between LAR and the risk of LVA across the three cohorts (overall P> 0.05). The predictive capacity of LAR for assessing LVA risk exceeded that of both lactate dehydrogenase and albumin (P< 0.05). Subgroup analyses further reinforced the robustness of these findings.

**Conclusion:**

An elevated LAR was independently associated with an increased risk of LVA development in patients with STEMI who underwent primary PCI.

## Introduction

Left ventricular aneurysm (LVA) is characterized by the outward expansion of infarcted myocardial tissue during both systole and diastole (1). The majority of LVA develop as a result of acute transmural myocardial infarction (MI) and are associated with a poor prognosis (2). These ventricular aneurysms are frequently linked to complications such as congestive heart failure, stroke, and ventricular arrhythmia (3, 4), which collectively contribute to increased mortality and morbidity rates in this patient population. The incidence of LVA formation has been reported to be as high as 7.6% among patients with coronary artery disease and 28.0% among those who have experienced an MI (2). Therefore, identifying the risk factors associated with LVA is essential for the development of preventive strategies.

Lactate dehydrogenase (LDH) is an intracellular enzyme integral to glycolysis, facilitating the conversion of pyruvate to lactic acid. Prior research has identified LDH as a biomarker indicative of disease activity and tissue damage, with significant associations to cardiovascular diseases. Wu et al. established that elevated LDH levels are independently associated with increased all-cause mortality in the U.S. general population with metabolic syndrome (5). Furthermore, in arsenic-endemic regions of southwestern Taiwan, LDH has been recognized as an independent predictor of cardiovascular disease mortality (6). A prospective study involving 109,632 incident dialysis patients revealed that LDH levels exceeding 280 U/L were linked to a heightened risk of both all-cause and cardiovascular mortality (7). Albumin, synthesized in the liver, is essential for binding and transport functions, maintaining plasma oncotic pressure, and reducing inflammatory responses (8). Feng et al. demonstrated that low baseline serum albumin levels are independently associated with decreased 4-year survival rates in patients with heart failure and severe secondary mitral regurgitation (9). Additionally, hypoalbuminemia was linked to increased in-hospital mortality and emerged as an independent predictor of long-term mortality in individuals with acute heart failure (10).

Recently, the lactate dehydrogenase to albumin ratio (LAR) has emerged as a novel indicator of inflammation, attracting considerable scholarly attention. For example, a retrospective cohort study indicated that LAR is associated with poor prognosis in patients with sepsis-associated acute kidney injury (11). Furthermore, a positive correlation has been observed between LAR and both in-hospital mortality and postoperative complications in patients undergoing isolated coronary artery bypass grafting (12). Additionally, Hu et al. demonstrated that an elevated LAR is linked to an increased risk of 30-day mortality in patients with acute pulmonary embolism (13).

Despite the growing interest in utilizing LAR as a prognostic marker, there is a conspicuous lack of studies, to the best of our knowledge, that have explored the relationship between LAR and LVA formation in patients experiencing acute ST-elevation myocardial infarction (STEMI). This study aims to evaluate the predictive value of LAR for the risk of LVA formation in the Chinese population.

## Methods

### Study design and population

The data for this study were collected from three medical institutions: the Central Hospital of Wuhan, Renmin Hospital of Wuhan University, and Central China Fuwai Hospital. The cohort was divided into three independent groups corresponding to the three recruitment centers. This multi-center design was implemented to validate the robustness, consistency, and generalizability of the study findings, and to reduce potential bias associated with single-center research. Ethical approval for the study was granted by the Review Boards of the aforementioned institutions. The study was conducted in compliance with the principles set forth in the Declaration of Helsinki. Written informed consent was obtained from all participants.

**Figure 1:**
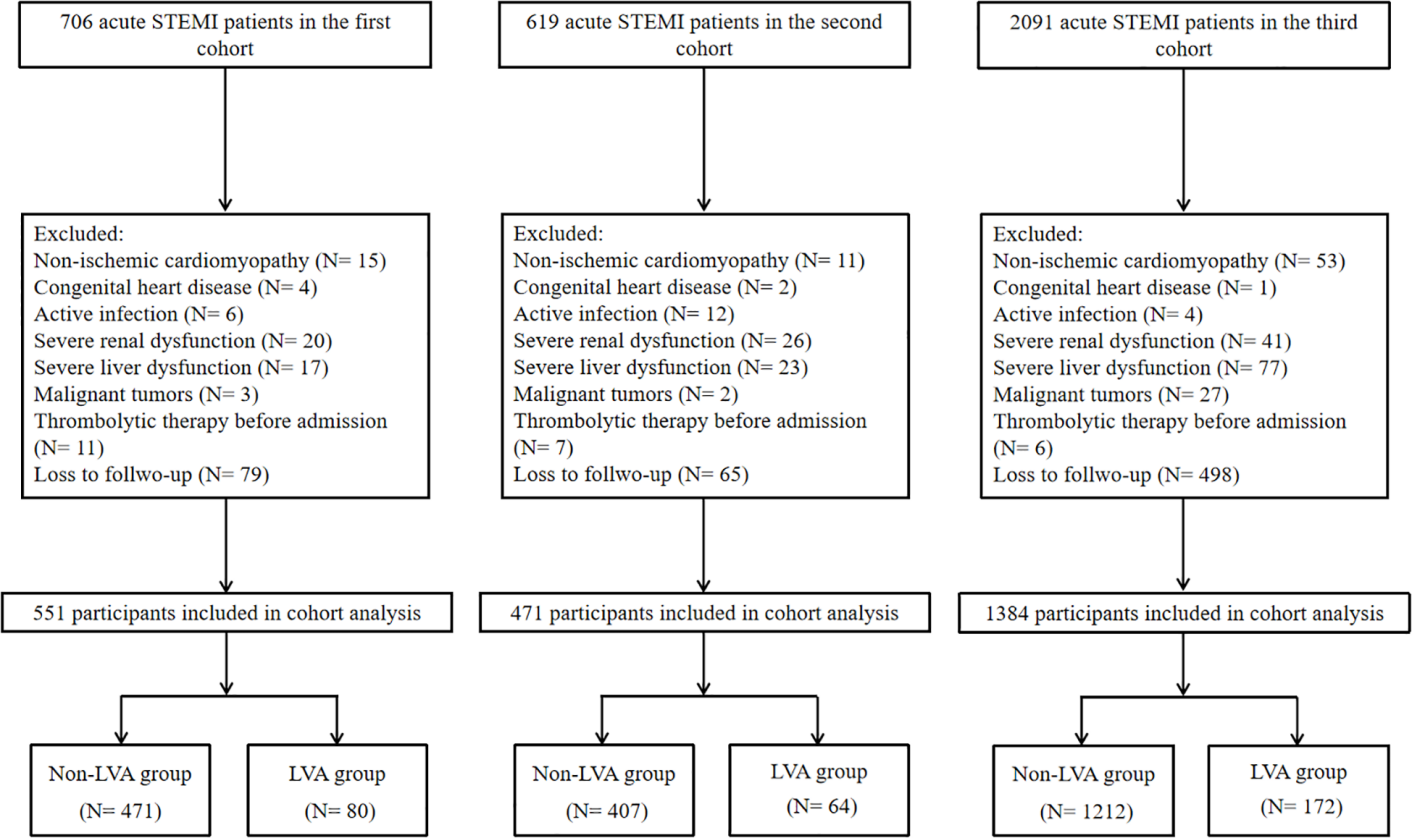
The flowchart of patient’s enrollment. LVA, Left ventricular aneurysm.

In the first cohort, a total of 706 consecutive patients diagnosed with acute ST-elevation myocardial infarction (STEMI) and treated with primary percutaneous coronary intervention (PCI) between December 2018 and February 2023 at the Central Hospital of Wuhan were enrolled. In the second cohort, 619 consecutive patients diagnosed with acute STEMI and treated with primary PCI at Renmin Hospital of Wuhan University between July 2018 and June 2022 were enrolled. Additionally, we recruited 2091 consecutive acute STEMI patients who underwent primary PCI at Central China Fuwai Hospital from 2018 to 2025. The diagnosis of acute STEMI was established in accordance with the Fourth Universal Definition of Myocardial Infarction (14). Exclusion criteria encompassed cases of non-ischemic cardiomyopathy (including hypertrophic and dilated types), congenital heart defects, ongoing infections, renal or hepatic failure, malignant neoplasms, individuals with a life expectancy of less than one year, those who received thrombolytic therapy prior to hospital admission, and patients lost to follow-up. Finally, the analyses included 551 patients in the first cohort, 471 patients in the second cohort, and 1,384 patients in the third cohort (**Figure 1**).

### Echocardiography

Upon admission, all patients underwent two-dimensional transthoracic echocardiography (TTE), which was subsequently repeated at the end of the first and sixth months of the follow-up period. The diagnosis of LVA was established using TTE in accordance with the protocol outlined in the Coronary Artery Surgery Study (CASS) (15). The criteria for diagnosing LVA included: (I) bulging of the left ventricular wall during diastole and systole, which showed either akinesia or dyskinesia; (II) a clear distinction of the infarcted segment; and (III) a lack of trabeculation in the involved segment.

### Covariates

The study incorporated covariates encompassing both sociodemographic and clinical characteristics evaluated at baseline. Sociodemographic variables comprised age (in years), sex (male or female), and smoking status (yes or no). Clinical characteristics included hypertension, diabetes, medications prescribed at discharge, results from angiographic evaluations, and various hematological test indicators. The diagnostic criteria for hypertension, diabetes mellitus, and smoking history have been previously established (16). Demographic and clinical data were extracted from the electronic medical record system by physicians who were blinded to the study’s objectives. Fasting venous blood samples from the elbow were collected from all participants within 24 hours of hospital admission (during the acute phase of STEMI, prior to primary PCI) for analysis. This time frame was selected to capture acute-phase biochemical changes (e.g., myocardial necrosis and systemic inflammation) that drive subsequent left ventricular remodeling and LVA formation. The hematological indicators assessed included white blood cell count (WBC), red blood cell count (RBC), hemoglobin, glycated hemoglobin (HbA1c), albumin, lactate dehydrogenase (LDH), Uric acid, serum creatinine, high-density lipoprotein (HDL), low-density lipoprotein (LDL), D-dimer, C reactive protein, N-terminal pro b-type natriuretic peptide (NT-proBNP).

### Statistical analysis

Continuous variables were characterized using either the mean and standard deviation or the median and interquartile range, contingent upon the normality of the data distribution. Categorical variables were expressed as frequencies and corresponding percentages. To evaluate differences among groups, one-way analyses of variance were employed for normally distributed data, Kruskal-Wallis tests for non-normally distributed data, and chi-square tests for categorical data. We divided the population equally into four groups based on the quartiles of LAR. Multivariate logistic regression analysis was conducted to explore the association between LAR and the risk of LVA development. The association analyses were adjusted using three models: Model 1 was an unadjusted model; Model 2 adjusted for age, sex; Model 3 further adjusted for hypertension, diabetes, smoking status, the use of Aspirin, Clopidogrel/Ticagrelorand, Statin, Beta blocker, Angiotensin converting enzyme inhibitor/angiotensin receptor blocker (ACEI/ARB), multiple vessel disease, Left anterior descending artery (LAD) as the Culprit vessel, post-procedural TIMI flow <3, use of drug-eluting stent, balloon dilation, and door-to-balloon time (DTB). Restricted cubic splines (RCS) analysis was performed to assess the nonlinear relationship between LAR and the risk of LVA. Analyses of subgroups were conducted to determine if the relationship between LAR and the risk of LVA varied according to factors such as sex, age, hypertension (Yes/no), diabetes (Yes/no), smoking status (Yes/no), LVEF (<50/≥ 50%), and LAD as the Culprit vessel (Yes/no), multiple vessel disease (Yes/no), the use of β-blockers, and use of ACEI/ARB. To evaluate the predictive ability of different variables in forecasting LVA, receiver operating characteristic (ROC) curve analyses were also utilized.

The statistical evaluations for this research were performed utilizing SPSS version 26 (IBM Corporation, Armonk, NY, USA). All comparisons were assessed as two-sided, with the criterion for statistical significance established at p < 0.05.

## Results

### General characteristics

The baseline characteristics of the three cohorts are presented in **Table 1**. The three cohorts exhibited generally comparable baseline characteristics, with minor differences attributable to real-world clinical variations across centers. The mean age of participants was 61.2 ± 12.9 years in the first cohort, 60.5 ± 12.2 years in the second cohort, and 60.4 ± 12.7 years in the third cohort. Across all cohorts, the proportion of female participants was lower compared to male participants, with percentages of 19.78% in the first cohort, 16.14% in the second cohort, and 24.13% in the third cohort.

**Table 1:** Descriptive characteristics of the study population.

Characteristics	First cohort				Second cohort				Third cohort			
	Whole cohort	Non-LVA patients	LVA patients	P-value	Whole cohort	Non-LVA patients	LVA patients	P-value	Whole cohort	Non-LVA patients	LVA patients	P-value
	N= 551	N= 471	N= 80		N= 471	N= 407	N= 64		N= 1384	N= 1212	N= 172	
Age, years, mean (SD)	61.2 ± 12.9	60.5 ± 12.8	65.1 ± 12.9	0.003	60.5 ± 12.2	60.1 ± 12.2	63.1 ± 12.1	0.067	60.4 ± 12.7	59.8 ± 12.6	64.8 ± 12.0	<0.001
Female, n (%)	109 (19.78)	87 (18.47)	22 (27.50)	0.061	76 (16.14)	59 (14.50)	17 (26.56)	0.015	334 (24.13)	263 (21.70)	71 (41.28)	<0.001
LVEF, (%)	54.15 ± 8.52	56.00 ± 7.09	43.24 ± 8.08	<0.001	48.96 ± 7.77	50.53 ± 6.80	39.76 ± 6.64	<0.001	48.12 ± 11.73	49.12 ± 11.66	41.03 ± 9.65	<0.001
Hypertension, n (%)	300 (54.45)	256 (54.35)	44 (55.00)	0.914	260 (55.20)	229 (56.27)	31 (48.44)	0.242	673 (48.63)	582 (48.02)	91 (52.91)	0.23
Diabetes, n (%)	176 (31.94)	147 (31.21)	29 (36.25)	0.371	138 (29.30)	116 (28.50)	22 (34.38)	0.337	369 (26.66)	319 (26.32)	50 (29.07)	0.445
Smoking, n (%)	298 (54.08)	263 (55.84)	35 (43.75)	0.045	269 (57.11)	237 (58.23)	32 (50.00)	0.216	726 (52.46)	650 (53.63)	76 (44.19)	0.02
WBC (×10^9^/L)	9.73 (7.77-12.13)	9.74 (7.81-12.05)	9.64 (7.70-12.43)	0.717	11.26 (9.51-13.83)	11.00 (9.38-13.73)	12.67 (10.45-14.41)	0.005	8.45 (6.54-11.33)	8.36 (6.52-11.02)	9.37 (6.80-12.68)	0.004
RBC (×10^9^/L)	4.47 ± 0.65	4.47 ± 0.66	4.42 ± 0.64	0.496	4.58 ± 0.61	4.61 ± 0.57	4.41 ± 0.76	0.05	4.65 ± 11.01	4.71 ± 11.76	4.18 ± 0.92	0.552
Hemoglobin (g/L)	138.43 ± 21.40	139.09 ± 21.58	134.56 ± 19.98	0.08	140.72 ± 18.74	141.88 ± 17.75	133.38 ± 22.93	0.006	136.94 ± 31.12	137.46 ± 29.52	133.27 ± 40.56	0.098
HbA1c, (%)	6.00 (5.60-6.90)	6.00 (5.60-6.90)	6.10 (5.70-7.60)	0.18	6.10 (5.70-7.40)	6.10 (5.70-7.40)	6.05 (5.62-8.00)	0.915	5.83 (5.48-7.01)	5.81 (5.46-7.03)	5.93 (5.56-6.89)	0.042
Albumin, (g/L)	41.03 ± 5.32	41.40 ± 5.21	38.86 ± 5.46	<0.001	39.13 ± 2.96	39.22 ± 3.01	38.57 ± 2.56	0.102	41.27 ± 8.45	41.43 ± 8.34	40.13 ± 9.13	0.059
LDH, (U/L)	312 (202-572)	284 (199-534)	471 (244-740)	<0.001	458 (295-685)	444 (281-656)	654 (397-884)	<0.001	325 (212- 663)	310 (210-614)	447 (251-1001)	<0.001
Uric acid (umol/L)	371 (308-438)	373 (308-439)	352 (309-425)	0.323	373 (312-446)	370 (311437)	401 (314-477)	0.102	318- (258-393)	316 (256-388)	333 (273-458)	0.015
Serum creatinine (umol/L)	82.15 ± 54.45	81.95 ± 56.37	83.36 ± 41.63	0.83	78.38 ± 58.47	77.37 ± 60.79	84.81 ± 40.58	0.344	86.44 ± 75.50	85.92 ± 78.59	90.10 ± 48.44	0.497
HDL (mmol/L)	0.98 (0.84-1.14)	0.98 (0.83-1.13)	0.98 (0.84-1.19)	0.598	0.99 (0.84-1.19)	1.00 (0.85-1.19)	0.94 (0.80-1.17)	0.269	0.95 (0.81-1.13)	0.95 (0.81-1.12)	0.96 (0.82-1.14)	0.925
LDL (mmol/L)	3.12 ± 1.08	3.09 ± 1.07	3.30 ± 1.10	0.106	2.94 ± 0.97	2.94 ± 0.96	2.92 ± 0.98	0.891	2.39 ± 2.23	2.39 ± 2.36	2.34 ± 0.90	0.766
D-dimer (ug/mL)	0.38 (0.23-0.65)	0.36 (0.22-0.61)	0.50 (0.29-0.99)	0.002	0.39 (0.21-0.81)	0.35 (0.20-0.69)	0.83 (0.40-2.11)	<0.001	0.48 (0.25-1.10)	0.45 (0.24-1.00)	0.87 (0.43-2.10)	<0.001
C reactive protein (mg/L)	0.70 (0.23-1.93)	0.60 (0.22-1.64)	1.98 (0.40-4.08)	<0.001	2.57 (0.84-7.36)	2.40 (0.84-6.10)	6.70 (1.38-29.61)	<0.001	6.01 (2.35-30.05)	5.36 (2.29-25.50)	14.98 (3.44-65.45)	<0.001
NT-proBNP (pg/mL)	586 (213-1722)	481 (201-1317)	3336 (952-7633)	<0.001	362 (113-1433)	314 (103-1116)	2287 (250-5133)	<0.001	1435 (538-3320)	1273 (481-2945)	2584 (1418-4875)	<0.001
Aspirin	551 (100.0)	471 (100.0)	80 (100.0)	>0.99	471 (100.0)	407 (100.0)	64 (100.0)	>0.99	1384 (100.0)	1212 (100.0)	172 (100.0)	>0.99
Clopidogrel/Ticagrelor	551 (100.0)	471 (100.0)	80 (100.0)	>0.99	471 (100.0)	407 (100.0)	64 (100.0)	>0.99	1384 (100.0)	1212 (100.0)	172 (100.0)	>0.99
Statin	551 (100.0)	471 (100.0)	80 (100.0)	>0.99	471 (100.0)	407 (100.0)	64 (100.0)	>0.99	1384 (100.0)	1212 (100.0)	172 (100.0)	>0.99
Beta blocker	474 (86.03)	407 (86.41)	67 (83.75)	0.526	415 (88.11)	362 (88.94)	53 (82.81)	0.159	1338 (96.68)	1173 (96.78)	165 (95.93)	0.56
ACEI/ARB	405 (73.50)	336 (71.34)	69 (86.25)	0.005	365 (77.49)	308 (75.68)	57 (89.06)	0.017	933 (67.41)	826 (68.15)	107 (62.21)	0.12
Culprit artery-LAD	289 (52.45)	221 (46.92)	68 (85.00)	<0.001	283 (60.08)	224 (55.04)	59 (92.19)	<0.001	1114 (80.49)	954 (78.71)	160 (93.02)	<0.001
proximal	188 (65.05)	145 (65.61)	43 (63.24)	0.807	210 (74.20)	163 (72.77)	47 (79.66)	0.55	810 (72.71)	681 (71.38)	129 (80.62)	0.051
mid	88 (30.45)	67 (30.32)	21 (30.88)		69 (24.38)	57 (25.45)	12 (20.34)		296 (26.57)	266 (27.88)	30 (18.75)	
distal	13 (4.50)	9 (4.07)	4 (5.88)		4 (1.41)	4 (1.79)	0 (0.00)		8 (0.72)	7 (0.73)	1 (0.62)	
Culprit artery-LCX	62 (11.25)	60 (12.74)	2 (2.50)	<0.001	54 (11.46)	52 (12.78)	2 (3.12)	<0.001	109 (7.88)	105 (8.66)	4 (2.33)	<0.001
proximal	26 (41.94)	25 (41.67)	1 (50.00)	>0.99	23 (42.59)	23 (44.23)	0 (0.00)	0.18	49 (44.95)	46 (43.81)	3 (75.00)	0.371
mid	31 (50.00)	30 (50.00)	1 (50.00)		26 (48.15)	25 (48.08)	1 (50.00)		32 (29.36)	32 (30.48)	0 (0.00)	
distal	5 (8.06)	5 (8.33)	0 (0.00)		5 (9.26)	4 (7.69)	1 (50.00)		28 (25.69)	27 (25.71)	1 (25.00)	
Culprit artery-RCA	200 (36.30)	190 (40.34)	10 (12.50)	<0.001	134 (28.45)	131 (32.19)	3 (4.69)	<0.001	161 (11.63)	153 (12.62)	8 (4.65)	<0.001
proximal	87 (43.50)	81 (42.63)	6 (60.00)	0.44	75 (55.97)	74 (56.49)	1 (33.33)	0.34	68 (42.24)	62 (40.52)	6 (75.00)	0.203
mid	87 (43.50)	83 (43.68)	4 (40.00)		44 (32.84)	43 (32.82)	1 (33.33)		73 (45.34)	71 (46.41)	2 (25.00)	
distal	26 (13.00)	26 (13.68)	0 (0.00)		15 (11.19)	14 (10.69)	1 (33.33)		20 (12.42)	20 (13.07)	0 (0.00)	
Post-procedural TIMI flow <3	27 (4.90)	21 (4.46)	6 (7.50)	0.376	12 (2.55)	10 (2.46)	2 (3.12)	>0.99	45 (3.25)	38 (3.14)	7 (4.07)	0.518
Use of drug-eluting stent	505 (91.65)	431 (91.51)	74 (92.50)	0.767	449 (95.33)	388 (95.33)	61 (95.31)	>0.99	1342 (96.97)	1177 (97.11)	165 (95.93)	0.398
Balloon dilation	547 (99.27)	468 (99.36)	79 (98.75)	0.467	463 (98.30)	400 (98.28)	63 (98.44)	>0.99	1364 (98.55)	1196 (98.68)	168 (97.67)	0.489
DTB, min	68 (50-86)	67 (48-85)	77 (59-92)	0.01	64 (41-85)	62 (39-84)	79 (50-96)	<0.001	65 (45-83)	63 (44-82)	76 (58-92)	<0.001
Multiple vessel disease, (%)	376 (68.24)	315 (66.88)	61 (76.25)	0.10	306 (64.97)	267 (65.60)	39 (60.94)	0.47	821 (59.32)	724 (59.74)	97 (56.40)	0.404

WBC, White blood cell count; RBC, Red blood cell count; HbA1c, glycated hemoglobin; LDH, Lactate dehydrogenase; HDL; high-density lipoprotein; LDL, low-density lipoprotein; NT-proBNP, N-terminal pro b-type natriuretic peptide; LVEF, left ventricular ejection fraction; ACEI/ARB, Angiotensin converting enzyme inhibitor/angiotensin receptor blocker; LAD, Left anterior descending artery; LCX, Left circumflex artery; RCA, Right coronary artery; DTB, door-to-balloon time; LVA, left ventricular aneurysm.

Levels of LDH, D-dimer, C-reactive protein, NT-proBNP, and DTB were significantly elevated in patients with LVA compared to those without LVA (P < 0.05). Conversely, LVEF was higher in non-LVA patients than in LVA patients. Furthermore, the incidence of LAD as the culprit vessel was more prevalent in LVA patients compared to non-LVA patients.

Subsequently, the population was stratified into four equal groups based on the quartiles of the LAR. **Tables 2**–**4** delineate the baseline characteristics of the participants according to these LAR quartiles. The incidence rates of LVA for quartiles Q1, Q2, Q3, and Q4 were 8.03%, 12.32%, 12.32%, and 25.36% in the first cohort; 5.13%, 12.71%, 11.86%, and 24.58% in the second cohort; and 8.38%, 10.98%, 12.14%, and 18.21% in the third cohort, respectively. Across all cohorts, individuals in the highest LAR quartile (Q4) demonstrated elevated levels of WBC, LDH, D-dimer, C-reactive protein, and NT-proBNP. In contrast, LVEF levels were significantly lower in participants within the LAR quartile Q4 compared to those in the other quartiles (P < 0.05).

**Table 2:** Characteristics of the first cohort according to the quartiles of LAR.

Characteristics	LAR				P-value
	Q1 (< 4.79)	Q2 (≥ 4.79, < 7.47)	Q3 (≥ 7.47, < 14.33)	Q4 (≥ 14.33)	
Participants, No	137	138	138	138	
Age, years, mean (SD)	59.82 ± 12.09	63.08 ± 11.96	61.85 ± 14.35	59.86 ± 12.77	0.094
Female, n (%)	17 (12.41)	28 (20.29)	32 (23.19)	32 (23.19)	0.081
LVEF, (%)	57.02 ± 7.82	55.01 ± 8.15	54.46 ± 7.99	50.11 ± 8.65	<0.001
Hypertension, n (%)	73 (53.28)	83 (60.14)	73 (52.90)	71 (51.45)	0.473
Diabetes, n (%)	51 (37.23)	43 (31.16)	37 (26.81)	45 (32.61)	0.321
Smoking, n (%)	83 (60.58)	72 (52.17)	63 (45.65)	80 (57.97)	0.062
WBC (×10^9^/L)	9.18 (6.80-11.67)	8.89 (7.47-11.05)	9.88 (8.20-12.02)	11.05 (8.92-13.64)	<0.001
RBC (×10^9^/L)	4.54 ± 0.56	4.40 ± 0.75	4.44 ± 0.61	4.50 ± 0.67	0.275
Hemoglobin (g/L)	143.62 ± 21.34	134.89 ± 23.45	137.11 ± 19.57	138.14 ± 20.28	0.006
HbA1c, (%)	6.10 (5.70-6.90)	6.00 (5.65-6.80)	5.90 (5.50-6.70)	6.00 (5.62-7.30)	0.463
Albumin, (g/L)	43.98 ± 4.82	40.60 ± 5.07	40.21 ± 4.58	39.34 ± 5.61	<0.001
LDH, (U/L)	168 (155-189)	237 (213-272)	412 (353-490)	824 (675-1046)	<0.001
Uric acid (umol/L)	373 (311-433)	365 (315-427)	381 (304-454)	363 (307-440)	0.851
Serum creatinine (umol/L)	81.87 ± 56.32	84.31 ± 40.23	78.50 ± 36.33	83.95 ± 76.25	0.804
HDL (mmol/L)	0.97 (0.82-1.08)	1.00 (0.86-1.16)	0.95 (0.83-1.10)	1.00 (0.85-1.19)	0.248
LDL (mmol/L)	3.08 ± 1.04	3.07 ± 0.97	3.11 ± 1.09	3.20 ± 1.21	0.742
D-dimer (ug/mL)	0.33 (0.22-0.49)	0.40 (0.23-0.68)	0.36 (0.22-0.67)	0.42 (0.28-0.78)	0.004
C reactive protein (mg/L)	0.50 (0.21-0.89)	0.71 (0.21-2.06)	0.68 (0.20-2.32)	1.10 (0.40-3.59)	<0.001
NT-proBNP (pg/mL)	299 (152-557)	609 (183-1215)	572 (286-2350)	1297 (482-2861)	<0.001
Aspirin	137 (100.0)	138 (100.0)	138 (100.0)	138 (100.0)	>0.99
Clopidogrel/Ticagrelor	137 (100.0)	138 (100.0)	138 (100.0)	138 (100.0)	>0.99
Statin	137 (100.0)	138 (100.0)	138 (100.0)	138 (100.0)	>0.99
Beta blocker	118 (86.13)	119 (86.23)	118 (85.51)	119 (86.23)	0.998
ACEI/ARB	96 (70.07)	104 (75.36)	101 (73.19)	104 (75.36)	0.723
Culprit artery-LAD	68 (49.64)	67 (48.55)	69 (50.00)	85 (61.59)	0.035
proximal	43 (63.24)	43 (64.18)	43 (62.32)	59 (69.41)	0.928
mid	21 (30.88)	22 (32.84)	22 (31.88)	23 (27.06)	
distal	4 (5.88)	2 (2.99)	4 (5.80)	3 (3.53)	
Culprit artery-LCX	11 (8.03)	16 (11.59)	15 (10.87)	20 (14.49)	0.035
proximal	3 (27.27)	7 (43.75)	9 (60.00)	7 (35.00)	0.594
mid	7 (63.64)	7 (43.75)	6 (40.00)	11 (55.00)	
distal	1 (9.09)	2 (12.50)	0 (0.00)	2 (10.00)	
Culprit artery-RCA	58 (42.34)	55 (39.86)	54 (39.13)	33 (23.91)	0.035
proximal	23 (39.66)	29 (52.73)	18 (33.33)	17 (51.52)	0.072
mid	30 (51.72)	22 (40.00)	26 (48.15)	9 (27.27)	
distal	5 (8.62)	4 (7.27)	10 (18.52)	7 (21.21)	
Post-procedural TIMI flow <3	6 (4.38)	14 (10.14)	2 (1.45)	5 (3.62)	0.007
Use of drug-eluting stent	126 (91.97)	123 (89.13)	127 (92.03)	129 (93.48)	0.617
Balloon dilation	135 (98.54)	138 (100.00)	137 (99.28)	137 (99.28)	0.481
DTB, min	67 (49-86)	66 (49-84)	68 (47-91)	71 (52-85)	0.727
Multiple vessel disease, (%)	89 (64.96)	97 (70.29)	93 (67.39)	97 (70.29)	0.739
LVA, (%)	11 (8.03)	17 (12.32)	17 (12.32)	35 (25.36)	<0.001

WBC, White blood cell count; RBC, Red blood cell count; HbA1c, glycated hemoglobin; LDH, Lactate dehydrogenase; HDL; high-density lipoprotein; LDL, low-density lipoprotein; NT-proBNP, N-terminal pro b-type natriuretic peptide; LAR, lactate dehydrogenase to albumin ratio; LVEF, left ventricular ejection fraction; ACEI/ARB, Angiotensin converting enzyme inhibitor/angiotensin receptor blocker; LAD, Left anterior descending artery; LCX, Left circumflex artery; RCA, Right coronary artery; DTB, door-to-balloon time; LVA, left ventricular aneurysm.

**Table 3:** Characteristics of the second cohort according to the quartiles of LAR.

Characteristics	LAR				P-value
	Q1 (< 7.31)	Q2 (≥ 7.31, < 11.66)	Q3 (≥ 11.66, < 17.47)	Q4 (≥ 17.47)	
Participants, No	117	118	118	118	
Age, years, mean (SD)	58.67 ± 11.16	63.08 ± 12.84	61.10 ± 11.40	59.16 ± 12.84	0.021
Female, n (%)	13 (11.11)	30 (25.42)	16 (13.56)	17 (14.41)	0.014
LVEF, (%)	52.78 ± 6.89	50.60 ± 7.51	47.78 ± 7.18	44.90 ± 7.23	<0.001
Hypertension, n (%)	68 (58.12)	68 (57.63)	64 (54.24)	60 (50.85)	0.652
Diabetes, n (%)	34 (29.06)	34 (28.81)	37 (31.36)	33 (27.97)	0.949
Smoking, n (%)	78 (66.67)	62 (52.54)	62 (52.54)	67 (56.78)	0.095
WBC (×10^9^/L)	10.19 (9.11-12.27)	11.16 (9.13-13.29)	10.79 (9.46-13.15)	13.10 (10.86-15.68)	<0.001
RBC (×10^9^/L)	4.58 ± 0.52	4.50 ± 0.65	4.61 ± 0.56	4.64 ± 0.68	0.308
Hemoglobin (g/L)	139.64 ± 16.22	138.76 ± 19.81	141.43 ± 18.16	143.04 ± 20.42	0.302
HbA1c, (%)	6.30 (5.80-7.40)	6.40 (5.80-7.80)	6.10 (5.60-7.30)	5.90 (5.60-6.75)	0.142
Albumin, (g/L)	39.63 ± 4.81	39.08 ± 1.76	39.03 ± 2.00	38.81 ± 2.22	0.186
LDH, (U/L)	230 (203-249)	364 (331-415)	556 (505-625)	873 (783-1115)	<0.001
Uric acid (umol/L)	372 (318-432)	363 (287-414)	385 (312-453)	381 (319-465)	0.071
Serum creatinine (umol/L)	78.40 ± 80.27	80.57 ± 72.53	73.99 ± 28.05	80.58 ± 35.98	0.803
HDL (mmol/L)	0.97 (0.80-1.14)	1.00 (0.83-1.18)	1.00 (0.85-1.19)	1.01 (0.85-1.21)	0.197
LDL (mmol/L)	2.84 ± 0.85	2.93 ± 0.93	2.96 ± 1.03	3.01 ± 1.04	0.61
D-dimer (ug/mL)	0.30 (0.16-0.49)	0.34 (0.20-0.72)	0.41 (0.25-0.78)	0.66 (0.32-1.51)	<0.001
C reactive protein (mg/L)	1.74 (0.38-4.33)	2.34 (0.82-6.05)	2.56 (0.97-5.73)	6.00 (1.42-20.68)	<0.001
NT-proBNP (pg/mL)	168 (59-516)	382 (130-1216)	311 (117-1054)	1476 (247-3563)	<0.001
Aspirin	117 (100.0)	118 (100.0)	118 (100.0)	118 (100.0)	>0.99
Clopidogrel/Ticagrelor	117 (100.0)	118 (100.0)	118 (100.0)	118 (100.0)	>0.99
Statin	117 (100.0)	118 (100.0)	118 (100.0)	118 (100.0)	>0.99
Beta blocker	99 (84.62)	98 (83.05)	107 (90.68)	111 (94.07)	0.029
ACEI/ARB	88 (75.21)	87 (73.73)	96 (81.36)	94 (79.66)	0.451
Culprit artery-LAD	66 (56.41)	59 (50.00)	69 (58.47)	89 (75.42)	<0.001
proximal	44 (66.67)	37 (62.71)	56 (81.16)	73 (82.02)	0.032
mid	20 (30.30)	21 (35.59)	13 (18.84)	15 (16.85)	
distal	2 (3.03)	1 (1.69)	0 (0.00)	1 (1.12)	
Culprit artery-LCX	9 (7.69)	20 (16.95)	16 (13.56)	9 (7.63)	<0.001
proximal	5 (55.56)	8 (40.00)	5 (31.25)	5 (55.56)	0.23
mid	3 (33.33)	10 (50.00)	11 (68.75)	2 (22.22)	
distal	1 (11.11)	2 (10.00)	0 (0.00)	2 (22.22)	
Culprit artery-RCA	42 (35.90)	39 (33.05)	33 (27.97)	20 (16.95)	<0.001
proximal	28 (66.67)	23 (58.97)	13 (39.39)	11 (55.00)	0.132
mid	13 (30.95)	11 (28.21)	13 (39.39)	7 (35.00)	
distal	1 (2.38)	5 (12.82)	7 (21.21)	2 (10.00)	
Post-procedural TIMI flow <3	2 (1.71)	2 (1.69)	4 (3.39)	4 (3.39)	0.773
Use of drug-eluting stent	113 (96.58)	109 (92.37)	114 (96.61)	113 (95.76)	0.36
Balloon dilation	115 (98.29)	117 (99.15)	115 (97.46)	116 (98.31)	0.882
DTB, min	63 (37-83)	66 (39-87)	62 (42-83)	67 (43-89)	0.43
Multiple vessel disease, (%)	76 (64.96)	81 (68.64)	76 (64.41)	73 (61.86)	0.749
LVA, (%)	6 (5.13)	15 (12.71)	14 (11.86)	29 (24.58)	<0.001

WBC, White blood cell count; RBC, Red blood cell count; HbA1c, glycated hemoglobin; LDH, Lactate dehydrogenase; HDL; high-density lipoprotein; LDL, low-density lipoprotein; NT-proBNP, N-terminal pro b-type natriuretic peptide; LAR, lactate dehydrogenase to albumin ratio; LVEF, left ventricular ejection fraction; ACEI/ARB, Angiotensin converting enzyme inhibitor/angiotensin receptor blocker; LAD, Left anterior descending artery; LCX, Left circumflex artery; RCA, Right coronary artery; DTB, door-to-balloon time; LVA, left ventricular aneurysm.

**Table 4:** Characteristics of the third cohort according to the quartiles of LAR.

Characteristics	LAR				P-value
	Q1 (< 5.05)	Q2 (≥ 5.05, < 8.18)	Q3 (≥ 8.18, < 16.97)	Q4 (≥ 16.97)	
Participants, No	346	346	346	346	
Age, years, mean (SD)	58.51 ± 13.07	61.03 ± 13.00	61.68 ± 11.99	60.37 ± 12.44	0.007
Female, n (%)	80 (23.12)	87 (25.14)	80 (23.12)	87 (25.14)	0.856
LVEF, (%)	52.50 ± 11.19	50.57 ± 11.11	48.87 ± 10.05	40.52 ± 10.88	<0.001
Hypertension, n (%)	175 (50.58)	160 (46.24)	165 (47.69)	173 (50.00)	0.637
Diabetes, n (%)	89 (25.72)	88 (25.43)	85 (24.57)	107 (30.92)	0.22
Smoking, n (%)	184 (53.18)	180 (52.02)	187 (54.05)	175 (50.58)	0.816
WBC (×10^9^/L)	6.89 (5.72-8.55)	7.50 (6.06-8.91)	8.72 (7.00-10.90)	12.50 (9.99-15.11)	<0.001
RBC (×10^9^/L)	4.41 ± 0.57	4.36 ± 0.55	5.47 ± 21.99	4.34 ± 0.87	0.458
Hemoglobin (g/L)	137.01 ± 22.03	137.71 ± 30.54	134.47 ± 30.36	138.58 ± 39.14	0.342
HbA1c, (%)	5.83 (5.47-6.86)	5.83 (5.50-6.97)	5.78 (5.46-6.78)	5.89 (5.49-7.36)	0.164
Albumin, (g/L)	46.98 ± 10.21	41.25 ± 7.38	39.30 ± 6.79	37.53 ± 5.55	<0.001
LDH, (U/L)	173 (154-197)	253 (223-288)	439 (360-544)	1083 (803-1516)	<0.001
Uric acid (umol/L)	317 (263-377)	312 (260-397)	301 (239-374)	340 (265-445)	<0.001
Serum creatinine (umol/L)	83.01 ± 103.41	82.81 ± 55.81	84.24 ± 63.79	95.71 ± 69.70	0.071
HDL (mmol/L)	0.92 (0.82-1.05)	0.94 (0.80-1.09)	0.95 (0.81-1.12)	1.02 (0.84-1.20)	<0.001
LDL (mmol/L)	2.43 ± 4.22	2.35 ± 0.88	2.38 ± 0.83	2.39 ± 0.87	0.968
D-dimer (ug/mL)	0.31 (0.19-0.51)	0.43 (0.27-0.77)	0.58 (0.28-1.35)	0.95 (0.38-2.51)	<0.001
C reactive protein (mg/L)	2.88 (1.46-6.36)	4.11 (2.21-9.10)	10.28 (2.84-38.73)	38.98 (9.13-111.62)	<0.001
NT-proBNP (pg/mL)	594 (210-1527)	1203 (417-3053)	1590 (811-3313)	2605 (1455-4853)	<0.001
Aspirin	346 (100.0)	346 (100.0)	346 (100.0)	346 (100.0)	>0.99
Clopidogrel/Ticagrelor	346 (100.0)	346 (100.0)	346 (100.0)	346 (100.0)	>0.99
Statin	346 (100.0)	346 (100.0)	346 (100.0)	346 (100.0)	>0.99
Beta blocker	337 (97.40)	339 (97.98)	334 (96.53)	328 (94.80)	0.102
ACEI/ARB	259 (74.86)	233 (67.34)	235 (67.92)	206 (59.54)	<0.001
Culprit artery-LAD	281 (81.21)	275 (79.48)	272 (78.61)	286 (82.66)	0.856
proximal	197 (70.11)	196 (71.27)	190 (69.85)	227 (79.37)	0.005
mid	83 (29.54)	79 (28.73)	80 (29.41)	54 (18.88)	
distal	1 (0.36)	0 (0.00)	2 (0.74)	5 (1.75)	
Culprit artery-LCX	24 (6.94)	30 (8.67)	30 (8.67)	25 (7.23)	0.856
proximal	12 (50.00)	11 (36.67)	17 (56.67)	9 (36.00)	0.443
mid	4 (16.67)	11 (36.67)	8 (26.67)	9 (36.00)	
distal	8 (33.33)	8 (26.67)	5 (16.67)	7 (28.00)	
Culprit artery-RCA	41 (11.85)	41 (11.85)	44 (12.72)	35 (10.12)	0.856
proximal	20 (48.78)	11 (26.83)	14 (31.82)	23 (65.71)	0.018
mid	17 (41.46)	24 (58.54)	24 (54.55)	8 (22.86)	
distal	4 (9.76)	6 (14.63)	6 (13.64)	4 (11.43)	
Post-procedural TIMI flow <3	21 (6.07)	18 (5.20)	4 (1.16)	2 (0.58)	<0.001
Use of drug-eluting stent	340 (98.27)	331 (95.66)	333 (96.24)	338 (97.69)	0.157
Balloon dilation	338 (97.69)	341 (98.55)	341 (98.55)	344 (99.42)	0.301
DTB, min	64 (46-83)	66 (48-83)	65 (45-82)	65 (47-83)	0.915
Multiple vessel disease, (%)	203 (58.67)	207 (59.83)	210 (60.69)	201 (58.09)	0.9
LVA, (%)	29 (8.38)	38 (10.98)	42 (12.14)	63 (18.21)	<0.001

WBC, White blood cell count; RBC, Red blood cell count; HbA1c, glycated hemoglobin; LDH, Lactate dehydrogenase; HDL; high-density lipoprotein; LDL, low-density lipoprotein; NT-proBNP, N-terminal pro b-type natriuretic peptide; LAR, lactate dehydrogenase to albumin ratio; LVEF, left ventricular ejection fraction; ACEI/ARB, Angiotensin converting enzyme inhibitor/angiotensin receptor blocker; LAD, Left anterior descending artery; LCX, Left circumflex artery; RCA, Right coronary artery; DTB, door-to-balloon time; LVA, left ventricular aneurysm.

### Correlation of LAR with LVA formation

**Table 5** presents a comprehensive summary of the association between LAR quantiles and the risk of LVA, as determined through logistic regression analysis. When treated as a continuous variable, LAR was significantly associated with the risk of LVA after full adjustment across all cohorts. In the first cohort, individuals in the fourth quartile (Q4) demonstrated a markedly increased risk of LVA compared to those in the first quartile (Q1) across all models: model 1 (OR = 3.89, 95% CI = 1.88 - 8.04, P< 0.001), model 2 (OR = 3.9, 95% CI = 1.87 - 8.14, P< 0.001), and model 3 (OR = 3.63, 95% CI = 1.65 - 7.96, P = 0.001). Similarly, participants in the LAR Q4 were associated with an increased risk of LVA in both the second cohort (OR = 6.01, 95% CI = 2.22 - 16.26, P< 0.001) and the third cohort (OR = 2.41, 95% CI = 1.47 - 3.96, P< 0.001) following full adjustment.

**Table 5:** Logistic regression analysis results for the association between LAR and the risk of LVA.

Cohorts	LVA	Model 1		Model 2		Model 3	
		OR (95%CI)	P-value	OR (95%CI)	P-value	OR (95%CI)	P-value
First cohort	LAR, Continuous	1.05 (1.02 - 1.07)	<0.001	1.05 (1.03 - 1.08)	<0.001	1.04 (1.01 - 1.07)	0.003
	LAR, Categories						
	Q1	Referrence		Referrence		Referrence	
	Q2	1.61 (0.72 - 3.58)	0.243	1.45 (0.65 - 3.25)	0.365	1.70 (0.72 - 4.01)	0.229
	Q3	1.61 (0.72 - 3.58)	0.243	1.46 (0.65 - 3.28)	0.356	1.72 (0.73 - 4.05)	0.217
	Q4	3.89 (1.88 - 8.04)	<0.001	3.90 (1.87 - 8.14)	<0.001	3.63 (1.65 - 7.96)	0.001
Second cohort	LAR, Continuous	1.06 (1.04 - 1.09)	<0.001	1.06 (1.03 - 1.09)	<0.001	1.07 (1.04 - 1.10)	<0.001
	LAR, Categories						
	Q1	Referrence		Referrence		Referrence	
	Q2	2.69 (1.01 - 7.21)	0.048	2.24 (0.83 - 6.09)	0.113	2.81 (0.95 - 8.30)	0.062
	Q3	2.49 (0.92 - 6.72)	0.072	2.37 (0.87 - 6.43)	0.09	2.45 (0.83 - 7.25)	0.105
	Q4	6.03 (2.40 - 15.16)	<0.001	5.98 (2.37 - 15.11)	<0.001	6.01 (2.22 - 16.26)	<0.001
Third cohort	LAR, Continuous	1.01 (1.01 - 1.02)	<0.001	1.01 (1.01 - 1.02)	<0.001	1.01 (1.01 - 1.02)	<0.001
	LAR, Categories						
	Q1	Referrence		Referrence		Referrence	
	Q2	1.35 (0.81 - 2.24)	0.249	1.26 (0.75 - 2.12)	0.373	1.23 (0.73 - 2.09)	0.432
	Q3	1.51 (0.92 - 2.49)	0.105	1.43 (0.86 - 2.38)	0.163	1.45 (0.86 - 2.44)	0.159
	Q4	2.43 (1.52 - 3.89)	<0.001	2.37 (1.48 - 3.82)	<0.001	2.41 (1.47 - 3.96)	<0.001

OR, odds ratio; CI, confidence interval.

Model 1: unadjusted.

Model 2: adjusted for age, sex.

Model 3: model 2+further adjusted for hypertension, diabetes, smoking status, the use of Aspirin, Clopidogrel/Ticagrelorand, Statin, Beta blocker, ACEI/ARB, multiple vessel disease, LAD as the Culprit vessel, post-procedural TIMI flow <3, use of drug-eluting stent, balloon dilation, and DTB.

**Figure 2:**
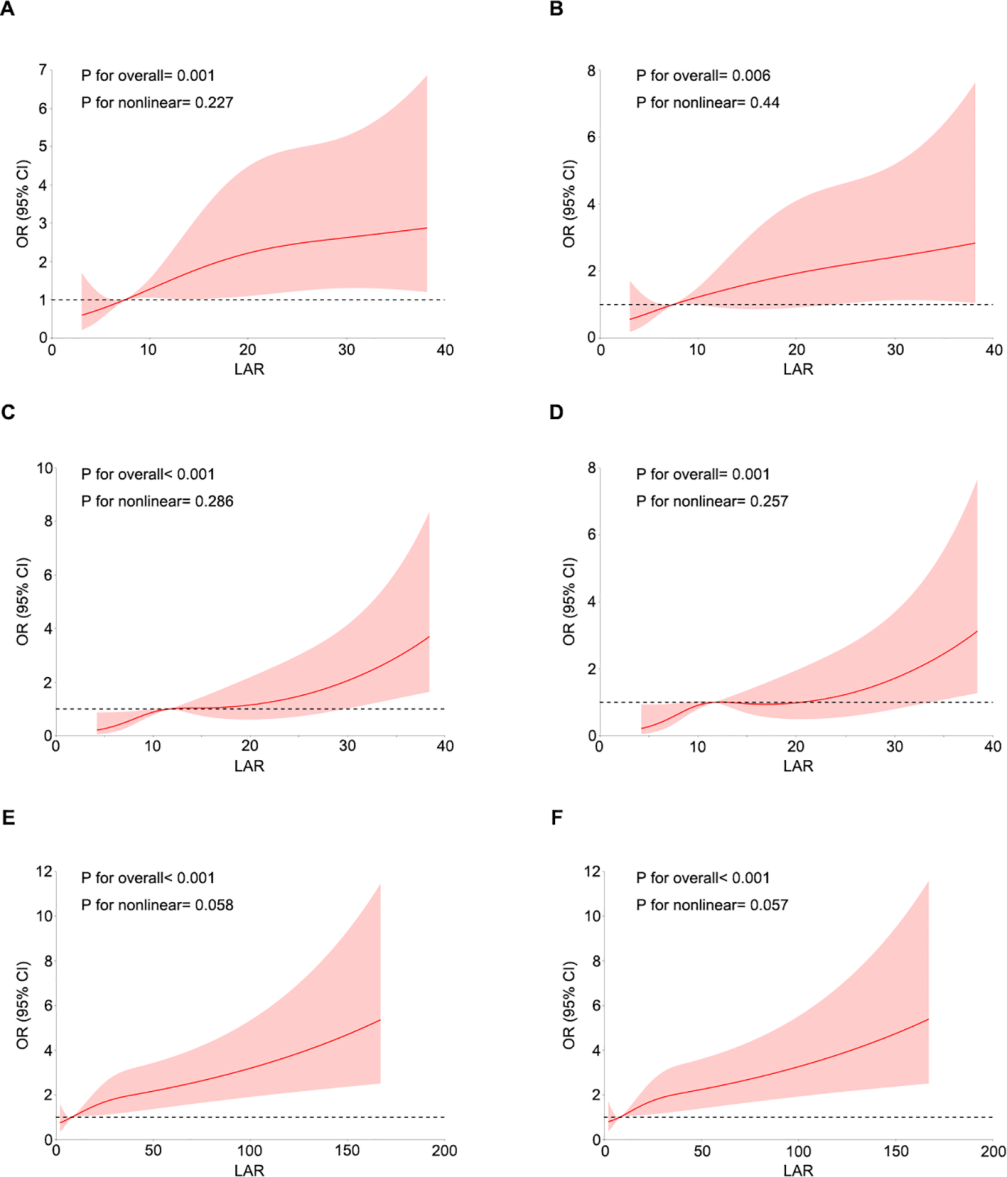
Restricted cubic spline curves for LVA by LAR using logistic regression analysis without **(A, C, E)** or with **(B, D, F)** adjustment for other covariates in the first **(A, B)**, second **(C, D)**, and third **(E, F)** cohort.

We employed a 4-knot restricted cubic spline (RCS) regression model to thoroughly investigate the dose-response relationship between the LAR and the risk of LVA development. As illustrated in **Figure 2**, LAR demonstrated a positive linear association with the risk of LVA, both before and after adjusting for potential confounding variables, including age, sex, hypertension, diabetes, smoking status, LAD as the culprit vessel, and multiple vessel disease, across all cohorts (nonlinear P > 0.05).

### Association between LAR and clinical index

**Figure 3:**
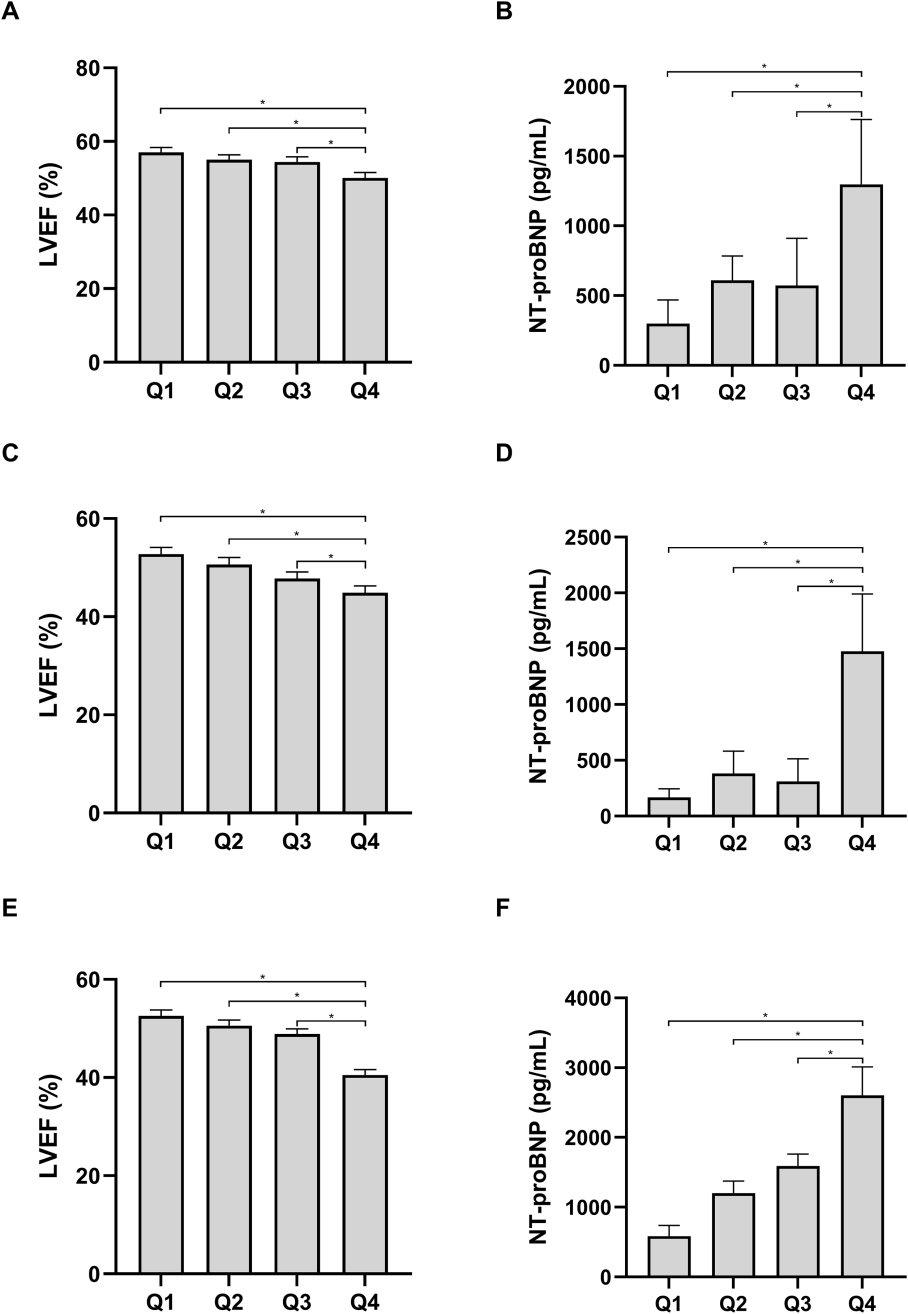
Comparison of LVEF and NT-proBNP across different LAR quartiles in the first **(A, B)**, second **(C, D)**, and third **(E, F)** cohort. LVEF, left ventricular ejection fraction; NT-proBNP, N-terminal pro b-type natriuretic peptide. *P< 0.05.

To assess the association between LAR and cardiac function, we conducted a comparative analysis of LVEF and NT-proBNP across various LAR quartiles. As illustrated in **Figure 3**, individuals in the Q4 LAR demonstrated significantly elevated levels of NT-proBNP and decreased LVEF relative to those in the other quartiles across all three cohorts (P < 0.05).

### Discriminative power analysis

**Figure 4:**
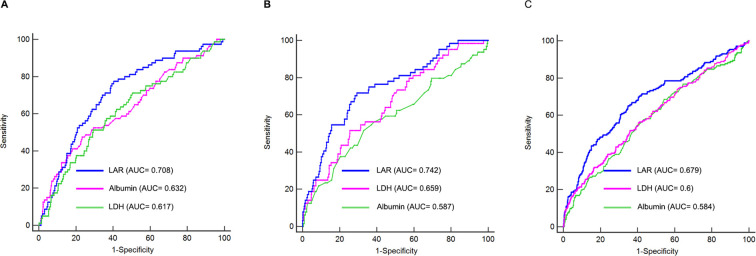
Receiver-operating characteristic curves for prediction of LVA formation in the first **(A)**, second **(B)**, and third **(C)** cohort. AUC, area under the curve.

We performed ROC analysis to evaluate and compare the predictive capabilities of albumin, LDH, and LAR in assessing the risk of LVA. In the first cohort, the mean AUCs value for albumin, LDH, and LAR were 0.632 (0.590 - 0.672), 0.617 (0.575 - 0.658), and 0.708 (0.668 - 0.746), respectively (**Table 6**, **Figure 4**). Notably, LAR exhibited significantly higher AUC values for predicting LVA formation compared to both albumin (P < 0.05) and LDH (P < 0.05). LAR also showed favorable specificity for LVA identification in each cohort (0.59, 0.71 and 0.65 for the first, second and third cohort, respectively), further supporting its value as a specific predictor for LVA. This increased predictive power of LAR over albumin and LDH was consistently observed in the second and third cohorts as well (**Table 6**, **Figure 4**).

**Table 6:** Analysis of the ROC curve for predictive power of left ventricular aneurysm formation.

Cohort	Variables	AUC	95% CI	Specificity	Sensitivity	Cut-off	p-Value#
The first cohort	LAR	0.708	0.668 - 0.746	0.59	0.78	12	–
	LDH	0.617	0.575 - 0.658	0.7	0.51	469	0.004
	Albumin	0.632	0.590 - 0.672	0.23	0.53	38.05	0.04
The second cohort	LAR	0.742	0.700 - 0.782	0.71	0.7	16.98	–
	LDH	0.659	0.613 - 0.702	0.74	0.52	652.5	0.01
	Albumin	0.587	0.541 - 0.632	0.33	0.48	38.45	0.011
The third cohort	LAR	0.679	0.654 - 0.704	0.65	0.65	14.98	–
	LDH	0.6	0.573 - 0.626	0.64	0.52	440.5	0.006
	Albumin	0.584	0.558 - 0.610	0.4	0.45	38.75	0.001

ROC, receiver operating characteristic; LAR, lactate dehydrogenase to albumin ratio; AUC, area under curve; CI, confidence interval.

p-Value#: comparison of LAR with other variables.

### Subgroup analysis

A subgroup analysis was performed to further evaluate the independent predictive value of the LAR in relation to LVA formation across a range of clinically relevant subgroups. In the first cohort, patients in the fourth quartile (Q4) demonstrated an elevated risk of LVA across all subgroups, except for smokers, those with LVEF < 50%, individuals without LAD as the culprit vessel, those without multiple vessel disease, and those not using β-blockers and ACEI/ARB, when compared to patients in the first quartile (Q1) (**Supplementary Table 1**). In the second cohort, an increased risk of LVA was observed in LAR Q4 among subgroups of patients aged ≥60 years, males, with or without hypertension, without diabetes, with or without smoking, with LVEF < 50%, with LAD as the culprit vessel, with multiple vessel disease, and those using β-blockers or ACEI/ARB (**Supplementary Table 2**). In the third cohort, the association between LAR Q4 and LVA was evident across all subgroups, except for patients aged ≥60 years, females, with hypertension, with diabetes, with LVEF < 50%, without LAD as the culprit vessel, and not using β-blockers (**Supplementary Table 3**). Furthermore, notable interactive effects between LVEF, ACEI/ARB use and LAR on the risk of LVA were observed in the third cohort (P for interaction < 0.05) (**Supplementary Table 3**).

## Discussion

To our knowledge, this is the first cohort study to explore the association between LAR and the risk of LVA formation in patients with STEMI who underwent primary PCI. In the present study, we found that an elevated LAR is associated with an increased risk of LVA formation. Subsequent analyses utilizing restricted cubic splines (RCS) corroborated the presence of a positive linear relationship between LAR and the likelihood of LVA, even after adjusting for a range of confounding variables. Moreover, the predictive capability of LAR for assessing the risk of LVA surpassed that of both albumin and LDH.

LDH is predominantly found in muscle and cardiac tissues, where it plays a crucial role in the production and dehydrogenation of lactic acid (17). Elevated serum LDH levels serve as an indicator of tissue degradation and are associated with various clinical conditions, such as inflammation (18), infection and sepsis (11), and hemolytic or hepatic disorders (19). Additionally, the significance of LDH in cardiovascular diseases has garnered increasing interest. An observational study involving 12,597 participants demonstrated that elevated LDH levels may correlate with arterial stiffness and a heightened 10-year risk of cardiovascular disease (20). A retrospective analysis of 109,632 individuals undergoing hemodialysis revealed that those with LDH levels exceeding 280 U/L experienced significantly higher rates of all-cause and cardiovascular mortality (7). Moreover, in arsenic-endemic regions of southwestern Taiwan, LDH has been identified as an independent predictor of cardiovascular disease mortality (6). In our study, LDH levels were significantly elevated in the LVA group compared to the non-LVA group, corroborating the strong association between LDH and cardiovascular diseases. Conversely, an inverse relationship exists between albumin levels and cardiovascular disease. In patients with advanced atherosclerosis and severe peripheral artery disease, low serum albumin was correlated with an increased risk of major adverse cardiac events (21). Feng et al. demonstrated that low baseline serum albumin levels are independently associated with decreased 4-year survival rates in patients with heart failure and severe secondary mitral regurgitation (9). Additionally, a low serum albumin concentration (<3.5 g/dL) may predict poor prognosis in patients with stable coronary heart disease (22).

The distinct roles of LDH and albumin in cardiovascular events imply that the LAR may provide enhanced predictive capability for cardiovascular diseases. Previous studies have consistently demonstrated a robust association between LAR and cardiovascular incidents. For instance, a retrospective cohort study revealed that LAR is linked to poor prognosis in patients with sepsis-associated acute kidney injury (11). Additionally, a positive correlation has been identified between LAR and both in-hospital mortality and postoperative complications in patients undergoing isolated coronary artery bypass grafting (12). Moreover, research by Hu et al. indicated that an elevated LAR is associated with an increased risk of 30-day mortality in patients with acute pulmonary embolism (13). In our current study, we similarly observed that a higher LAR was significantly correlated with an increased risk of LVA in patients experiencing acute ST-elevation myocardial infarction. Furthermore, individuals in the Q4 LAR quartile exhibited elevated levels of NT-proBNP and reduced levels of LVEF, compared to those in other LAR quartiles across three cohorts. These findings suggest that an elevated LAR is indicative of more severe coronary artery stenosis and myocardial injury. Furthermore, LAR demonstrated a superior predictive ability for LVA compared to LDH and albumin when considered separately. Overall, these findings underscore the significant role of LAR in cardiovascular diseases, aligning with and supporting prior research in the field (12, 13).

Numerous prior studies have explored the predictors of LVA in patients with AMI. For instance, Ran et al. identified the monocyte to high-density lipoprotein cholesterol ratio as an independent predictor of LVA development in patients with STEMI who underwent PCI (23). However, their investigation was limited by its single-center design and lack of extensive validation. In contrast, our study draws conclusions from three independent cohorts, thereby offering more robust and compelling evidence. Similarly, Wang et al. determined that post-PCI platelet count serves as an independent predictor of LVA in patients with anterior STEMI (24). Nevertheless, their study was constrained by a sample size of only 281 patients. Our research, involving a larger and multi-center cohort, provides more reliable findings. Furthermore, a retrospective cohort study involving 1823 STEMI patients identified female sex, peak NT-proBNP, the time between the onset of pain and balloon time, presence of QS-waves on initial electrocardiogram, RWMA of left ventricular anterior wall and apex as independent predictors of LVA formation (2). Although this research highlighted several risk factors for LVA, its broad scope did not allow for a focused analysis of any specific risk factor, which may limit the credibility of its conclusions. In contrast, our study provides a comprehensive and detailed examination of the association between LAR and the risk of LVA. By accounting for confounding risk factors, analyzing RCS curves, and conducting subgroup analyses, our study offers more robust and convincing findings. Notably, LAR exhibits both biological and clinical specificity for LVA prediction in STEMI patients, and distinct advantages over the above-reported predictors. Biologically, LAR is a pathogenically specific indicator: elevated LDH directly reflects myocardial tissue necrosis and local inflammatory response (the core pathological basis of LVA formation after transmural myocardial infarction), while reduced albumin indicates impaired systemic inflammatory regulation and myocardial tissue repair capacity—their ratio integrates two key pathophysiological dimensions closely and specifically linked to LVA development, rather than a single pathological process reflected by most existing biomarkers (e.g., monocyte-to-HDL cholesterol ratio for inflammation only, post-PCI platelet count for platelet function only). Clinically, LAR showed good specificities for LVA identification across our three multi-center cohorts (0.59, 0.71 and 0.65 for the first, second and third cohort, respectively; **Table 6**), and its predictive value remained robust across most clinically relevant subgroups, confirming its clinical specificity for the STEMI population. Compared with existing predictors, LAR also has superior clinical practicability: it is calculated from two routine blood test indicators (LDH and albumin) that are widely detected in all levels of medical institutions, without the need for specialized or invasive examinations required for some other predictors. Additionally, our multi-center validation with 2406 eligible patients provides more rigorous and generalizable evidence for LAR’s predictive value, in contrast to most existing studies limited by single-center design or small sample sizes.

As an observational study, we cannot definitively establish a causal relationship between LAR and LVA; however, several biologically plausible mechanisms may explain the observed association, which are outlined below: 1. LDH is a cytoplasmic enzyme abundantly expressed in cardiomyocytes. Upon irreversible ischemic injury, LDH is rapidly released into the circulation, and its serum level correlates positively with infarct size and the extent of myocardial necrosis (25). Elevated LDH reflects greater initial myocardial damage, which is a well-established determinant of subsequent left ventricular remodeling and aneurysm formation. 2. Albumin is a negative acute-phase protein; its concentration declines during systemic inflammation due to increased capillary permeability and reduced hepatic synthesis. In the setting of acute myocardial infarction, hypoalbuminemia reflects heightened inflammatory and oxidative stress responses (26). Low albumin levels have been associated with increased neutrophil infiltration, higher levels of pro-inflammatory cytokines (e.g., IL-6, TNF-α), and impaired antioxidant capacity, all of which can exacerbate myocardial injury and promote adverse remodeling. 3. The combination of extensive myocardial necrosis (high LDH) and exaggerated inflammatory response (low albumin) may synergistically accelerate left ventricular remodeling. High LDH indicates a large infarct burden, which triggers a robust inflammatory cascade, while low albumin fails to counteract oxidative stress and matrix degradation. This milieu promotes activation of matrix metalloproteinases (MMPs), particularly MMP-2 and MMP-9, leading to extracellular matrix degradation, thinning of the infarcted wall, and progressive ventricular dilation (27). Furthermore, persistent inflammation and oxidative stress can stimulate fibroblast proliferation and collagen deposition, contributing to wall stiffness and ultimately aneurysm formation. It is important to emphasize that these mechanistic pathways are speculative and derived from experimental and clinical studies on LDH, albumin, and cardiac remodeling. Future experimental studies—including animal models and *in vitro* investigations—are warranted to establish causality and to elucidate the precise molecular mechanisms linking LAR to LVA development.

Based on the pathophysiological mechanisms linking elevated LAR to LVA, several targeted interventions could be evaluated in future clinical trials. First, early and rapid revascularization to minimize infarct size and reduce myocardial necrosis (the main driver of high LDH) may lower LAR and attenuate LVA risk. Second, anti-inflammatory and cardioprotective therapies, such as low-dose colchicine or statin therapy, could be tested to suppress excessive inflammation, improve albumin status, and mitigate adverse remodeling. Third, intensified heart failure therapy (e.g., early uptitration of beta-blockers, ACEI/ARB, or MRAs) may help prevent ventricular dilation and aneurysm formation in high-LAR patients. Fourth, serial monitoring of LAR during hospitalization could be used to risk-stratify patients and guide personalized intensive follow-up. These interventions are clinically feasible and directly target the biological pathways underlying the association between LAR and LVA.

This study provides compelling evidence supporting the predictive value of the LAR for assessing the risk of LVA formation in patients with AMI across three cohorts. However, our findings should be interpreted with caution due to several limitations. First, the inclusion and exclusion criteria were relatively broad to enhance the external generalizability of the findings, as the study aimed to reflect real-world clinical scenarios of STEMI patients undergoing primary PCI. While this may have introduced some heterogeneity in the study population, we mitigated this potential impact through strict statistical adjustments (e.g., adjusting for 15 clinically relevant confounding factors in Model 3 of multivariate logistic regression) and subgroup analyses across key clinical variables (age, gender, LVEF, etc.), which confirmed the robustness of LAR’s predictive value. Future studies with more standardized and stringent criteria are still needed to further validate our findings in more homogeneous populations. Second, the diagnosis of LVA was based on echocardiographic assessment without core laboratory adjudication. We acknowledge that echocardiography is operator-dependent, and the reproducibility of LVA diagnosis may be suboptimal, which could lead to misclassification bias. To minimize this limitation: (1) All participating centers strictly adhered to the Coronary Artery Surgery Study (CASS) criteria for LVA diagnosis to ensure consistency in diagnostic standards; (2) LVA diagnosis was performed by experienced cardiologists blinded to the study’s objectives and LAR levels; (3) Transthoracic echocardiography (TTE) was repeated at 1 and 6 months of follow-up to confirm persistent LVA, reducing the risk of false-positive diagnosis from acute-phase myocardial stunning. Nevertheless, cardiac magnetic resonance (CMR) imaging offers higher spatial resolution and better reproducibility for LVA detection, and future studies should consider using CMR or implementing core laboratory reading to standardize LVA identification. Third, due to the retrospective design of the study, selection bias may be present despite our efforts to minimize it. To address this: (1) We enrolled consecutive STEMI patients from three independent centers to reduce selection bias from single-center sampling; (2) We adjusted for a comprehensive set of confounding variables (including demographic, clinical, and laboratory indicators) in the multivariate regression models; (3) Sensitivity analyses (e.g., subgroup analyses and restricted cubic spline plots) confirmed the stability of the association between LAR and LVA. However, unmeasured or residual confounding cannot be entirely excluded, and prospective cohort studies with predefined inclusion criteria and standardized outcome ascertainment are warranted to validate our results. Fourth, although the study included a substantial sample size, it focused exclusively on baseline levels of LDH and albumin, thereby overlooking potential variations in LAR that might offer deeper insights into the underlying mechanisms. Future studies could dynamically monitor LAR levels during follow-up to explore its longitudinal association with LVA development. Fifth, the observational nature of this study precludes the establishment of a causal relationship between LAR and LVA formation. Future experimental studies—including animal models and *in vitro* investigations—are needed to elucidate the precise molecular mechanisms linking LAR to LVA and confirm causality. Lastly, our findings are derived from a cohort of middle-aged and elderly Chinese Han patients who experienced acute STEMI and underwent PCI treatment. The generalizability of the proposed LAR and the model to other ethnicities, patients with non-STEMI, or those treated with alternative strategies (e.g., thrombolytic therapy) requires further investigation.

## Conclusion

Our study evaluated the predictive value of LAR for the risk of LVA in patients with STEMI who underwent PCI. The findings indicate that an elevated LAR is independently correlated with an increased risk of LVA in this patient population. Further investigations into the role of LAR in LVA development are warranted, as they will enhance our understanding of the underlying pathophysiological mechanisms and contribute to the advancement of novel preventive and therapeutic approaches for this condition.

## Data Availability

The original contributions presented in the study are included in the article/**Supplementary Material**. Further inquiries can be directed to the corresponding authors.
